# Therapie der akuten diabetischen Stoffwechselentgleisungen bei Erwachsenen (Update 2023): Hyperglykämisch-hyperosmolare und ketoazidotische Stoffwechselentgleisung

**DOI:** 10.1007/s00508-023-02174-8

**Published:** 2023-04-20

**Authors:** Susanne Kaser, Harald Sourij, Martin Clodi, Bruno Schneeweiß, Anton N. Laggner, Anton Luger

**Affiliations:** 1 Department für Innere Medizin 1, Medizinische Universität Innsbruck, Innsbruck, Österreich; 2 Klinische Abteilung für Endokrinologie und Diabetologie, Universitätsklinik für Innere Medizin, Medizinische Universität Graz, Graz, Österreich; 3 Abteilung für Innere Medizin, Krankenhaus Barmherzige Brüder Linz, Linz, Österreich; 4 Intensivstation, Klinische Abteilung für Gastroenterologie und Hepatologie Universitätsklinik für Innere Medizin III, Medizinische Universität Wien, Wien, Österreich; 5 Universitätsklinik für Notfallmedizin, Medizinische Universität Wien, Wien, Österreich; 6 Klinische Abteilung für Endokrinologie und Stoffwechsel, Universitätsklinik für Innere Medizin III, Medizinische Universität Wien, Spitalgasse 23, 1090 Wien, Österreich

**Keywords:** Diabetische Ketoazidose, Hyperklämisch-hyperosmolare Stoffwechselentgleisung, Serum-Osmolalität, Anionenlücke, Pseudohyponatriämie, Diabetic ketoacidosis, Hyperglycemic hyperosmolar state, Serum osmolality, Anion gap, Pseudohyponatremia

## Abstract

Akute Stoffwechselentgleisungen können für Erwachsene in Abhängigkeit von ihrem Ausmaß lebensbedrohlich sein. Dementsprechend sind eine rasche umfassende Diagnostik und Therapie sowie eine enge Überwachung der Vitalparameter und Laborbefunde erforderlich. Bei der Therapie, die sich bei der ketoazidotischen (DKA) und hyperglykämisch-hyperosmolaren (HHS) Form nicht wesentlich unterscheidet, kommt dem Ausgleich des meist beträchtlichen Flüssigkeitsdefizits mit mehreren Litern einer physiologischen kristalloiden Lösung eine vorrangige Rolle zu. Bei den Elektrolyten ist insbesondere auf eine ausgeglichene Serum-Kalium-Konzentration zu achten. Normal-Insulin oder rasch wirksame Analoga können initial als i.v.-Bolus verabreicht werden, in der Folge jedoch kontinuierlich über einen Perfusor. Die Umstellung auf eine subkutane Insulintherapie soll erst bei ausgeglichenem Säure-Basen-Haushalt und zufriedenstellender Glykämie erfolgen.

## Abstract (en)

Diabetic ketoacidosis (DKA) and the hyperglycemic hyperosmolar state (HHS) represent potentially life-threatening situations in adults. Therefore, rapid comprehensive diagnostic and therapeutic measures with close monitoring of vital and laboratory parameters are required. The treatment of DKA and HHS is essentially the same and replacement of the mostly substantial fluid deficit with several liters of a physiological crystalloid solution is the first and most important step. Serum potassium concentrations need to be carefully monitored to guide its substitution. Regular insulin or rapid acting insulin analogues can be initially administered as an i.v. bolus followed by continuous infusion. Insulin should be switched to subcutaneous injections only after correction of the acidosis and stable glucose concentrations within an acceptable range.

Ketoazidotische Stoffwechselentgleisungen (DKA) sind wesentlich häufiger als hyperglykämisch-hyperosmolare (HHS). Erstere betreffen zu zwei Dritteln Menschen mit Typ-1-Diabetes und überwiegend jüngere Patienten im Alter von 18 bis 44 Jahren [1]. Die HHS tritt im Gegensatz dazu viel häufiger bei Menschen mit Typ-2-Diabetes auf und ist auch aufgrund des höheren Alters des betroffenen Kollektivs und der damit assoziierten häufigeren Komorbiditäten mit einer deutlich höheren Mortalität verbunden (weniger als 1 % bei der DKA und 5–20 % bei der HHS) [1, 2].

## Pathogenese, klinisches Bild und Befunde

Die Unterteilung der diabetischen Stoffwechselentgleisungen in DKA und HHS muss als grob schematisch betrachtet werden, da die DKA stets mit einer Azidose, oft aber nur mit einer mäßiggradigen Hyperglykämie und umgekehrt die HHS mit einem sehr hohen Blutzuckerwert, aber einer meist nur gering ausgeprägten Azidose verbunden ist (Tab. 1). Die DKA ist durch absolutes Fehlen von Insulin gekennzeichnet und infolgedessen fehlender Unterdrückung der Lipolyse und Verwertung freier Fettsäuren. Bei der HHS steht im Gegensatz dazu ein relativer Insulinmangel mit gesteigerter Glukoneogenese und beeinträchtigter peripherer Glukoseverwertung im Vordergrund. Im klinischen Alltag bestehen trotzdem weitgehend Überschneidungen, die HHS ist meist durch einen pH > 7,3 und ein Bikarbonat von > 18 mmol/l gekennzeichnet (Tab. 1). Aggravierend für die Ausbildung der Stoffwechselentgleisung sind zudem die Dehydrierung und der damit verbundene Anstieg der gegenregulatorischen Stresshormone Adrenalin, Noradrenalin und Kortisol [3–6]. Stets sollte an eine möglicherweise auslösende Ursache der Stoffwechselentgleisung gedacht werden, häufig ein Infekt, und eine ggf. erforderliche Diagnostik veranlasst werden. Weitere Ursachen für eine DKA sind häufig die Erstmanifestation eines Diabetes mellitus Typ 1, unterlassene Insulinapplikation oder Insulinpumpendefekte. Insbesondere bei der DKA kann die manchmal bestehende Pseudoperitonitis eine Herausforderung darstellen und muss bei der Indikationsstellung für eine Laparotomie unbedingt berücksichtigt werden.

**Table 1:** 

	DKA	HHS
Plasmaglukose (mg/dl)	> 250	> 600
Arterieller pH	< 7–7,3	> 7,3
Serumbikarbonat (mmol/l)	< 10–18	> 18
Harnketone	Positiv	Negativ oder schwach positiv
Serumketone (mmol/l)	3–> 8	< 0,6
Effektive Serumosmolalität	Variabel	> 320
Anionenlücke	> 10	Variabel
Bewusstseinslage	Klar–Koma	Stupor–Koma

Für das Auftreten einer Bewusstseinstrübung bzw. eines Komas ist vorwiegend die Hyperosmolalität verantwortlich. Bei einer Serumosmolarität < 320 mmol/l muss jedenfalls nach einer anderen Ursache für das Vorliegen des Komas gesucht werden, die ja auch die Ursache für die Stoffwechselentgleisung darstellen kann. Für die Beurteilung der *Serumosmolalität* ist die errechnete von größerer Bedeutung als die gemessene, wobei hier die Formel

$$\text{Serumosmolalit{\"a}t }(\mathrm{mmol}/\mathrm{l})=2\times \mathrm{Na}^{+}(\mathrm{mmol}/\mathrm{l})+\mathrm{K}^{+}(\mathrm{mmol}/\mathrm{l})+\text{Glukose }(\mathrm{mg}/\mathrm{dl})\colon 18$$

heranzuziehen ist, da BUN ungehindert zwischen Extra- und Intrazellulärraum diffundieren kann und in diesem Fall nicht berücksichtigt werden muss.

Die *Anionenlücke*, die sich anhand der Formel

$$\text{Anionenl{\"u}cke}=\mathrm{Na}^{+}(\mathrm{mmol}/\mathrm{l})-\left(\mathrm{CI}^{-}(\mathrm{mmol}/\mathrm{l})+{\mathrm{HCO}}_{3}^{-}(\mathrm{mmol}/\mathrm{l})\right)$$

errechnet [7–10], ist bei der DKA aufgrund der Erhöhung der *K*etone ebenfalls vergrößert. Dabei ist zu berücksichtigen, dass die Anionenlücke auch bei *U*rämie, durch *S*alicylate, *M*ethylalkohol/Äthanol, *A*ldehyde, bei *L*aktatazidose und durch Ä(*E*)thylenglycol vergrößert ist (Merkwort: *KUSMALE*).

Zu beachten ist ferner, dass in Abhängigkeit vom Ausmaß der Hyperglykämie auch eine *Pseudohyponatriämie* vorliegen kann: Pro 100 mg/dl über den Normbereich erhöhte Blutzuckerkonzentration ist die gemessene Serum-Natrium-Konzentration um 1,6 mmol/l niedriger [1].

## Therapie

Für die Therapie von vordringlicher Bedeutung ist zunächst der Ausgleich des Flüssigkeitsdefizits, da Insulin sonst nicht wirken kann. Durch die Hyperglykämie kommt es zu einer osmotischen Diurese, die zunächst durch eine „Autotransfusion“ von Flüssigkeit aus dem Intrazellulär- in den Extrazellulärraum ausgeglichen wird. Sie bleibt so lange bestehen, wie die Hyperglykämie besteht. Durchschnittliche Flüssigkeits- und Elektrolytdefizite bei DKA und HHS sind in Tab. 2 angeführt.

**Table 2:** 

	DKA	HHS
Flüssigkeit (l)	6	9
H_2_O (ml/kg)	100	100–200
Na (mmol/kg)	7–10	5–13
Cl (mmol/kg)	3–5	5–15
K (mmol/kg)	3–5	4–6
PO_4_ (mmol/kg)	5–7	3–7
Mg (mmol/kg)	1–2	1–2
Ca (mmol/kg)	1–2	1–2

In zahlreichen Publikationen wird eine Flüssigkeitssubstitution primär mit 0,9 % Kochsalzlösung empfohlen [1, 4, 5, 8–12]. Aufgrund der unphysiologischen Zusammensetzung (Natriumkonzentration 154 mmol/l, Chloridkonzentration 154 mmol/l) besteht aus pathophysiologischer Sicht aber das Risiko einer Verschlechterung der Azidose durch eine hyperchlorämische Azidose [13]. Besser geeignet für die Flüssigkeitssubstitution scheinen daher kristalloide Lösungen mit physiologischerer Zusammensetzung z. B. Elo-Mel® isoton (Tab. 3) zu sein. Es liegen auch Studien vor, die auf einen günstigeren klinischen Verlauf der diabetischen Ketoazidose bei Therapie mit einer kristalloiden Lösung mit einer dem Blutplasma ähnlicheren Elektrolyt-Zusammensetzung im Vergleich zu einer Therapie mit einer 0,9 % Kochsalzlösung hinweisen [14–17]. In der Regel sollte bei schweren Stoffwechselentgleisungen in der ersten halben Stunde bis Stunde 1 l Flüssigkeit verabreicht werden und auch in den weiteren 1 ½ bis 2 h jeweils etwa 1 l pro Stunde in Abhängigkeit von Hydratation, Diurese, Körpergewicht, kardialer und renaler Funktion sowie Elektrolyten. Falls ein zentraler Venenkatheter vorhanden ist, kann die Flüssigkeitssubstitution nach dem ZVD gegeben werden: < 0: 1000 ml/h, 0–3: 500 ml/h, 4–8: 250 ml/h, 9–12: 100 ml/h, > 12: keine Flüssigkeitsgabe.

**Table 3:** 

	Plasma	0,9 % NaCl	Elo-Mel® isoton	KADC	Ringer	Ringer-Laktat
Na	135–145	154	140	90	147	131
K	3,5–5,0	–	5	25	4	5,4
Ca	2,2–2,6	–	2,5	1,0	2,2	1,8
PO_4_	0,8–1,5	–	–	10	–	–
Mg	0,7–1,0	–	1,5	1,5	–	1
Cl	95–110	154	108	65	155	112
HCO_3_	21–26	–	Acetat 45	Malat 23	–	Laktat 28
Osmolalität	280–295	308	302	215	309	278

Auch auf die *Kaliumsubstitution* sollte von Anfang an größte Aufmerksamkeit gerichtet sein: Bei Kaliumkonzentrationen unter 3,3 mmol/l sollten vor Beginn einer Insulintherapie 20–40 mmol Kaliummalat (oder alternativ Kaliumchlorid) verabreicht werden, da sonst eine Aggravierung der Hypokaliämie durch den Shift von Kalium mit Insulin und Glukose von extrazellulär nach intrazellulär und durch den Ausgleich der Azidose verursacht werden kann und die Patienten einer zusätzlichen Gefährdung ausgesetzt werden. Bei Kaliumwerten zwischen 3,3 und 5,3 mmol/l sollten 20 mmol Kaliummalat zu jedem Liter Flüssigkeit zugegeben werden. Lediglich bei initialen Kaliumkonzentrationen von > 5,3 mmol/l und deutlichen Hinweisen für eine Hyperkaliämie (EKG: Bradykardie, SA Block oder höhergradiger AV Block, hohe, spitze T‑Zacken) empfiehlt es sich, zunächst mit der Kaliumsubstitution zuzuwarten [18] und kaliumfreie Elektrolytlösungen zu verwenden.

*Insulin*, meist ein kurz wirksames Analogon, kann initial als Bolus in einer Dosierung von 6 bis 8 Einheiten (oder 0,1 IE/kgKG) i.v. gegeben werden, in der Folge jedoch als kontinuierliche Infusion, die nach aktuellen Blutzuckerwerten angepasst werden muss (Tab. 4). Bei der DKA soll bei Blutzuckerwerten unter 200 mg/dl und bei der HHS bei Werten unter 250 mg/dl mit der Infusion einer 5 % Glukoselösung begonnen werden [8], um zu große und abrupte Abfälle der Glukose zu vermeiden und die weitere Insulininfusion zum Ketoazidoseausgleich ohne Hypoglykämie zu erlauben. Die Gefahr eines Hirnödems durch rasche Blutzuckerabfälle ist vorwiegend bei Kindern gegeben.

**Table 4:** 

Blutzuckerwerte	Insulindosis
< 80 mg/dl	Pause, Kontrolle nach einer halben Stunde
81–120 mg/dl	0,7 ml/h (0,7 E/h)
121–150 mg/dl	1 ml/h (1 E/h)
151–180 mg/dl	1,5 ml/h (1,5 E/h)
181–210 mg/dl	2 ml/h (2 E/h)
211–240 mg/dl	2,5 ml/h (2,5 E/h)
241–270 mg/dl	3 ml/h (3 E/h)
271–300 mg/dl	3,5 ml/h (3,5 E/h)
301–330 mg/dl	4 ml/h (4 E/h)
331–360 mg/dl	4,5 ml/h (4,5 E/h)
361–390 mg/dl	5 ml/h (5 E/h)
391–420 mg/dl	5,5 ml/h (5,5 E/h)
421–450 mg/dl	6 ml/h (6 E/h)

Bei Werten über 450 mg/dl kann eine Insulindosis von 8 E/h gewählt werden, darüber hinausgehende Dosen allenfalls in besonderen Ausnahmesituationen

N. B.: Sobald ein Patient/eine Patientin essen darf, sollten zusätzlich präprandial 1–1,5 E Insulinanalogon/BE verabreicht werden

Für die Gabe von *Bikarbonat* besteht keine Evidenz für einen positiven Effekt, es sind auch keine Studien zu erwarten, die den Benefit einer solchen Therapie bei pH-Werten < 7 nahelegen. Es besteht aber Übereinkunft, dass bei Patienten mit einem pH-Wert < 6,9 50–100 mmol Natriumbikarbonat (50–100 mL einer 8,4 % Lösung) in 200–400 ml H_2_O über 2 h verabreicht werden sollen [1]. Allerdings sollten die zur Flüssigkeitssubstitution eingesetzten Infusionslösungen ein organisches Salz enthalten, aus welchem Bikarbonat gebildet wird.

Für die Gabe von *Phosphat* gibt es keine Evidenz für positive Effekte.

Die Therapie von schweren diabetischen Stoffwechselentgleisungen sollte, wenn möglich, zunächst unter Monitoring auf einer Überwachungsstation erfolgen. Blutzuckerwerte müssen anfangs stündlich und im weiteren Verlauf 2‑ bis 3‑stündlich kontrolliert werden, um die Insulindosis anpassen zu können. Auch die Serumelektrolyte Natrium, Kalium, Chlorid und Phosphat ebenso wie Kreatinin, BUN und pH sollten anfangs in Abhängigkeit von den Ausgangswerten zunächst zumindest 2‑stündlich und dann 4‑stündlich kontrolliert werden. Da der venöse pH nur um 0,03 Einheiten niedriger als der arterielle ist, sind venöse Kontrollen des pH durchaus ausreichend, wenn keine andere Indikation für eine arterielle Blutgasanalyse gegeben ist.

Die Umstellung auf eine subkutane Insulintherapie sollte erst erfolgen, wenn die Azidose ausgeglichen ist und stabile Blutzuckerwerte in einem akzeptablen Bereich vorliegen. Der Ausgleich der Azidose beansprucht mehr Zeit als die Korrektur der Blutzuckerwerte.

## Interessenkonflikt

S. Kaser hat von folgenden Unternehmen, die z. T. auch fördernde Mitglieder der ÖDG sind, Forschungsunterstützungen und/oder Honorare erhalten: AstraZeneca, Boehringer Ingelheim, Eli Lilly, Merck Sharp & Dohme, Novartis, Novo Nordisk, Viatris. H. Sourij hat von folgenden Unternehmen, die auch fördernde Mitglieder der ÖDG sind, Forschungsunterstützungen und/oder Honorare erhalten: Amgen, AstraZeneca, Eli Lilly, Merck Sharp & Dohme, Novo Nordisk, Sanofi-Aventis, Takeda. M. Clodi hat von folgenden Unternehmen, die auch fördernde Mitglieder der ÖDG sind, Forschungsunterstützungen und/oder Honorare erhalten: AstraZeneca, Boehringer Ingelheim, Eli Lilly, Merck Sharp & Dohme, Novartis, Novo Nordisk, Sanofi-Aventis, Servier, Takeda. B. Schneeweiß, A.N. Laggner und A. Luger geben an, dass kein Interessenkonflikt besteht.
